# Factors associated with incidence of acute kidney injury: a Japanese regional population-based cohort study, the Shizuoka study

**DOI:** 10.1007/s10157-022-02310-0

**Published:** 2022-12-27

**Authors:** Hisashi Dote, Eiji Nakatani, Kiyoshi Mori, Akira Sugawara

**Affiliations:** Shizuoka Graduate University of Public Health, 4-27-2 Kita Ando, Aoi-ku, Shizuoka, Japan

**Keywords:** Acute kidney injury, Population-based cohort, Risk factors, Incidence

## Abstract

**Background:**

Acute kidney injury (AKI) is a globally critical issue. Most studies about AKI have been conducted in limited settings on perioperative or critically ill patients. As a result, there is little information about the epidemiology and risk factors of AKI in the general population.

**Methods:**

We conducted a population-based cohort study using the Shizuoka Kokuho Database. We included subjects with records of health checkup results. The observation period for each participant was defined as from the date of insurance enrollment or April 2012, whichever occurred later, until the date of insurance withdrawal or September 2020, whichever was later. Primary outcome was AKI associated with admission based on the ICD-10 code. We described the incidence of AKI and performed a multivariate analysis using potential risk factors selected from comorbidities, medications, and health checkup results.

**Results:**

Of 627,814 subjects, 8044 were diagnosed with AKI (incidence 251 per 100,000 person-years). The AKI group was older, with more males. Most comorbidities and prescribed medications were more common in the AKI group. As novel factors, statins (hazard ratio (HR) 0.84, 95% confidence interval (CI) 0.80–0.89) and physical activity habits (HR 0.79, 95% CI 0.75–0.83) were associated with reduced incidence of AKI. Other variables associated with AKI were approximately consistent with those from previous studies.

**Conclusions:**

The factors associated with AKI and the incidence of AKI in the general Japanese population are indicated. This study generates the hypothesis that statins and physical activity habits are novel protective factors for AKI.

**Supplementary Information:**

The online version contains supplementary material available at 10.1007/s10157-022-02310-0.

## Introduction

Acute kidney injury (AKI) is defined by a rapid rise in serum creatinine, a decrease in urine output, or both. The “Kidney Disease: Improving Global Outcomes (KDIGO)” diagnostic criteria and severity classification are used widely [1]. AKI is not only a poor prognostic factor in critically ill patients [2] but is also associated with worse long-term survival and renal outcome [3]. AKI is recognized as a critical issue globally, and the International Society of Nephrology has launched the 0by25 initiative, an international cross-sectional study to eliminate preventable deaths from AKI [4]. Patient-related risk factors for AKI have been identified, including nephrotoxic drugs (including drugs aimed to treat other conditions) and various comorbidities [5].

Although many cohort studies have been conducted to date, epidemiological information, such as the true incidence of AKI, is still unclear [6]. Although many studies have investigated the risk factors and prognostic implications of AKI, most have focused on a limited setting, such as critically ill patients, specific comorbidities, or the perioperative period [2, 7, 8]. Few studies conducted in the general population have investigated the effects of modifiable lifestyles.

Identifying the risk factors of AKI (especially modifiable risks) and informing citizens and healthcare providers may reduce the occurrence of AKI. Therefore, this study aims to describe the incidence of AKI associated with hospital admission in the general population and to explore the risk factors.

## Materials and methods

### Study design and data source

This population-based cohort study analyzed the data obtained from the Shizuoka Kokuho Database (SKDB) [9], an administrative claims database of health insurance subscribers in the municipalities of Shizuoka Prefecture, Japan. This database includes both National Health Insurance (those aged < 75 years) and Latter-Stage Elderly Medical Care System (those aged ≥ 75 years) subscribers. SKDB covers 8.5 years (April 1, 2012 to September 30, 2020) and includes 2,230,848 individuals, and contains basic information (sex, age, observation period, and reason for disenrollment, including death) about individuals, records of health checkup results, and dates of diagnosis and treatment based on the International Statistical Classification of Diseases and Related Health Problems, Tenth Revision (ICD-10), with medications prescribed.

### Participant population

The subjects were those registered in SKDB with records of health checkup results. The observation period for each participant was defined as from the date of insurance enrollment or April 2012, whichever occurred later, until the date of insurance withdrawal or September 2020, whichever was later.

We extracted the records of the first health checkup during the observation period. One year before the date of the first health checkup was defined as the baseline period. We extracted information about comorbidities and prescribed medications during the baseline period. We excluded participants who were on maintenance dialysis. Participants who had not received health checkups or who did not realize 1-year baseline periods were also excluded.

### Outcome and variables

The primary outcome was AKI associated with admission. We identified this outcome using the ICD-10 codes for AKI (N17, N19) [10–12] that are used for diagnosis during hospitalization. Our definition included community-acquired and hospital-acquired AKI. Comorbidities were extracted using ICD-10 codes for diagnoses equivalent to the Charlson Comorbidity Index [13]. Prescribed medications were extracted using the Anatomical Therapeutic Chemical Classification System. In selecting medications, we referred to previous studies and guidelines [14, 15]. We selected medications commonly used to treat chronic disease in the general population among those that might be associated with AKI as covariates. In addition, we extracted the following results from the health checkups: age, sex, body mass index (BMI), blood pressure, questionnaire responses about smoking habits and physical activity (“In your daily life, do you walk or do any equivalent amount of physical activity more than one hour a day?”), and results from laboratory examinations.

### Statistical analysis

Continuous and categorical variables were described as mean ± standard deviation and number (%), respectively. We used Fisher’s exact test to compare the proportions of binary variables and Student’s *t*-test to compare the continuous variables, and log-rank tests to evaluate differences in the cumulative incidence of AKI. We used a cause-specific proportional hazards model to adjust for confounders in the primary outcome. Death was considered as a competing event for AKI. Variables that were significant in the univariate model were included in the multivariate model. Complete case analysis was conducted to construct the multivariate model. Variables with Spearman’s correlation coefficients greater than 0.4 were used as covariates, whichever was considered more clinically important. Factors that were statistically significant in the multivariate model were defined as risk factors. We also performed a sensitivity analysis including interaction terms. Survival time variables were drawn as Kaplan–Meier curves, and log-rank tests were performed for comparisons between groups. The missing covariates used in the analysis did not occur completely randomly among all participants; therefore, we did not impute them. Statistical significance was defined as *P* < 0.05. We used JMP 16.2 (SAS Institute, Cary, NC, USA) and EZR version 1.55 (Saitama Medical Center, Jichi Medical University, Saitama, Japan) [16] for all statistical analyses.

## Results

### Characteristics of the patients

**Fig. 1:**
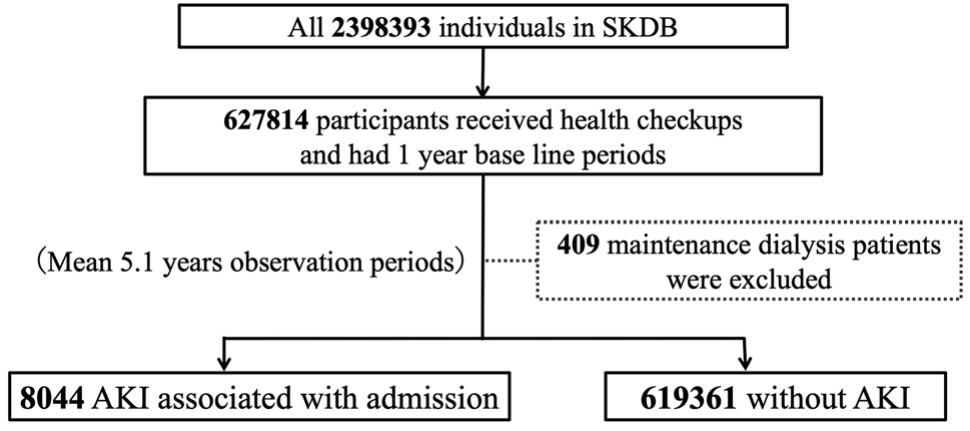
Flow diagram of our study. *SKDB* Shizuoka Kokuho Database, *AKI* acute kidney injury

Among all individuals in the SKDB, 627,814 were included in the analysis. Four hundred and nine maintenance dialysis patients were excluded. During the mean 5.1 years of observation, 8044 cases were diagnosed with AKI associated with hospital admission (Fig. 1).

Patients’ characteristics, divided into two groups according to AKI associated with admission (AKI group) or not (No AKI group), are shown in Table 1. The AKI group was older and comprised more males. BMI was higher in the AKI group, but the difference was minimal. Most comorbidities and prescribed medications were more common in the AKI group. The results of health checkups showed that base renal function (serum creatinine and estimated glomerular filtration rate (GFR)) was slightly worse in the AKI group. The AKI group also had a lower hemoglobin level. The number of habits with no physical activity was higher in the AKI group.

**Table 1:** Characteristics of the patients

Variable	Category or unit	AKI group	No AKI group	*P* value
		(*n* = 8044)	(*n* = 619,361)	
Age, mean (SD)	1 year	77.8 (9.0)	68.1 (11.2)	< 0.001
Age, *n* (%)	0 to < 40 years	3 (0.0)	4762 (0.8)	< 0.001
	40 to < 50 years	71 (0.9)	45,676 (7.4)	< 0.001
	50 to < 60 years	166 (2.1)	54,298 (8.8)	< 0.001
	60 to < 70 years	1231 (15.3)	233,171 (37.6)	< 0.001
	70 to < 80 years	2738 (34.0)	188,901 (30.5)	< 0.001
	≥ 80 years	3835 (47.7)	92,553 (14.9)	< 0.001
Sex	Male	4842 (60.2)	265,917 (42.9)	< 0.001
Comorbidities				
Congestive heart failure, *n* (%)	Presence	2615 (32.5)	55,072 (8.9)	< 0.001
Myocardial infarction, *n* (%)	Presence	449 (5.6)	9724 (1.6)	< 0.001
Peripheral vascular disease, *n* (%)	Presence	1834 (22.8)	64,733 (10.5)	< 0.001
Cerebrovascular disease, *n* (%)	Presence	2531 (31.5)	91,213 (14.7)	< 0.001
Dementia, *n* (%)	Presence	558 (6.9)	16,096 (2.6)	< 0.001
Chronic pulmonary disease, *n* (%)	Presence	2602 (32.3)	149,794 (24.2)	< 0.001
Rheumatic disease, *n* (%)	Presence	407 (4.9)	17,734 (2.9)	< 0.001
Peptic ulcer disease, *n* (%)	Presence	2600 (32.3)	118,709 (19.2)	< 0.001
Liver disease, *n* (%)	Presence	1919 (23.9)	101,026 (16.3)	< 0.001
Diabetes, *n* (%)	Presence	1440 (17.9)	34,374 (5.5)	< 0.001
Hypertension, *n* (%)	Presence	6674 (83.0)	295,166 (47.7)	< 0.001
Hemiplegia or paraplegia, *n* (%)	Presence	153 (1.9)	4821 (0.8)	< 0.001
Renal disease, *n* (%)	Presence	2510 (31.2)	12,225 (2.0)	< 0.001
Any malignancy, *n* (%)	Presence	1319 (16.4)	51,129 (8.3)	< 0.001
Medications				
ACE inhibitor, *n* (%)	Prescribed	601 (7.5)	16,631 (2.7)	< 0.001
ARB, *n* (%)	Prescribed	4334 (53.9)	150,430 (24.3)	< 0.001
MRA, *n* (%)	Prescribed	545 (6.8)	8335 (1.3)	< 0.001
CCB, *n* (%)	Prescribed	4885 (60.7)	191,099 (30.9)	< 0.001
β-Blocker, *n* (%)	Prescribed	1081 (13.4)	28,682 (4.6)	< 0.001
Diuretics, *n* (%)	Prescribed	2105 (26.2)	35,291 (5.7)	< 0.001
SGLT2 inhibitor, *n* (%)	Prescribed	15 (0.2)	2160 (0.3)	0.02
NSAIDs, *n* (%)	Prescribed	4261 (51.4)	228,337 (36.9)	< 0.001
Statin, *n* (%)	Prescribed	2656 (33.0)	157,807 (25.5)	< 0.001
Fibrate, *n* (%)	Prescribed	281 (3.5)	12,137 (2.0)	< 0.001
Gout suppressant, *n* (%)	Prescribed	2342 (29.1)	37,724 (6.1)	< 0.001
Result of health checkup				
Systolic blood pressure, mean (SD)	1 mmHg	134.27 (17.88)	129.40 (17.28)	< 0.001
AST, mean (SD)	1 U/L	24.36 (12.06)	24.16 (11.01)	0.12
ALT, mean (SD)	1 U/L	17.59 (11.90)	20.30 (13.64)	< 0.001
γ-GTP, mean (SD)	1 U/L	35.72 (56.70)	32.90 (46.78)	< 0.001
HDL-Chol, mean (SD)	1 mg/dL	55.38 (16.06)	62.38 (16.64)	< 0.001
LDL-Chol, mean (SD)	1 mg/dL	110.41 (31.48)	123.90 (31.24)	< 0.001
Serum creatinine, mean (SD)	1 mg/dL	1.30 (0.80)	0.76 (0.21)	< 0.001
Estimated GFR, mean (SD)	1 mL/min/1.73 m^2^	45.99 (19.74)	69.58 (15.45)	< 0.001
Hemoglobin, mean (SD)	1 g/dL	12.24 (1.87)	13.60 (1.47)	< 0.001
Questionnaire response				
Physical activity habits, *n* (%)	No	3813 (55.5)	275,905 (49.5)	< 0.001

The eGFR was calculated as follows: 194 × creatinine^−1.094^ × age^−0.287^ (× 0.739 [if women])

*AKI* acute kidney injury, *SD* standard deviation, *ACE* angiotensin-converting enzyme, *ARB* angiotensin receptor blocker, *MRA* mineralocorticoid receptor antagonist, *CCB* calcium channel blocker, *SGLT2* sodium-glucose transporter 2, *NSAIDs* nonsteroidal anti-inflammatory drugs, *AST* aspartate transaminase, *ALT* alanine aminotransferase, *GTP* glutamyl transpeptidase, *HDL* high-density lipoprotein, *LDL* low-density lipoprotein, *Chol* cholesterol, *GFR* glomerular filtration rate.

### Incidence of AKI associated with admission

**Fig. 2:**
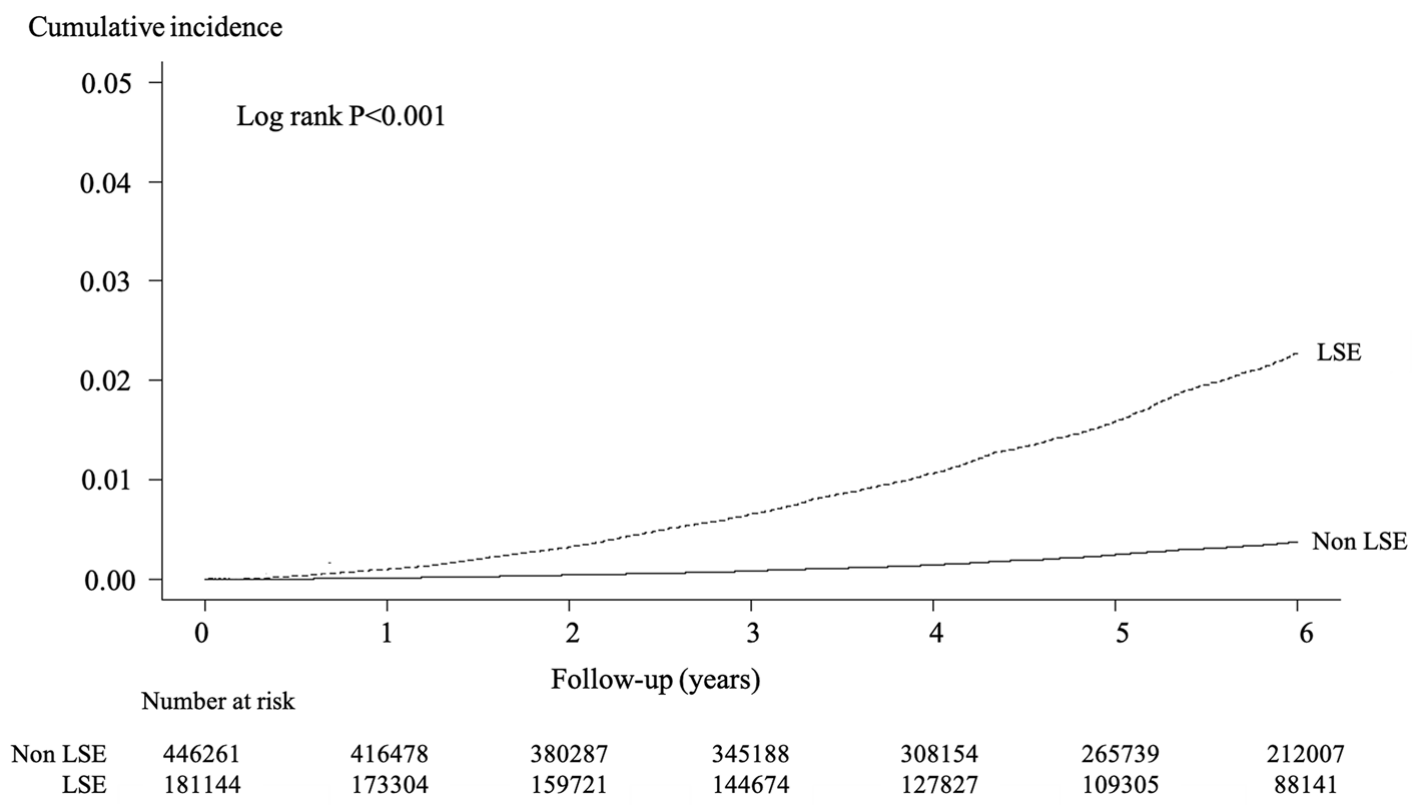
Cumulative incidence of acute kidney injury stratified by latter-stage elderly (LSE) or not

**Fig. 3:**
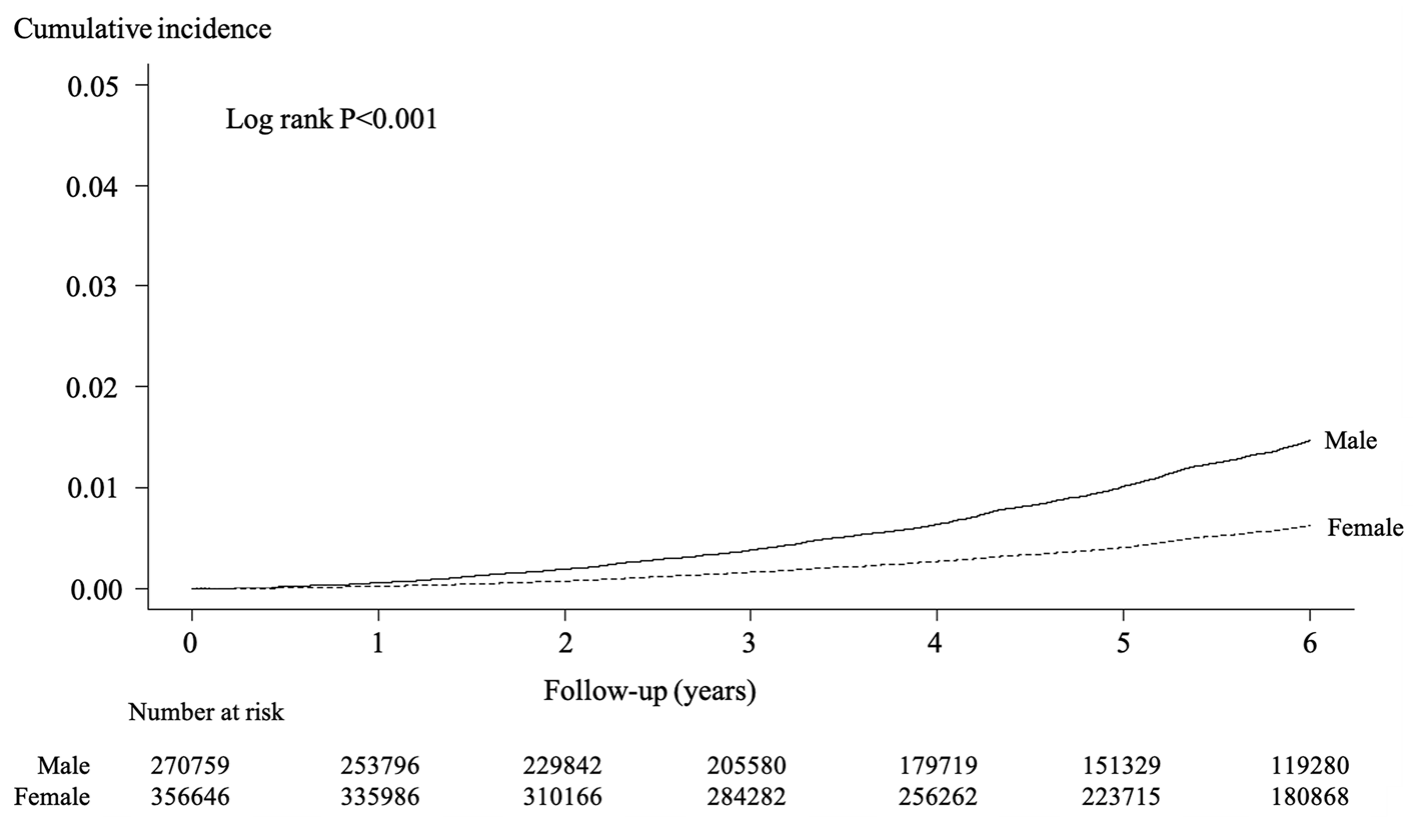
Cumulative incidence of acute kidney injury stratified by sex

The incidence of AKI was 251 per 100,000 person-years (mean 5.1 years of observation). The cumulative incidence of AKI is shown in Figs. 2, 3. In the stratified analysis, the incidence was higher in latter-stage older adults (Fig. 2) and males (Fig. 3).

### Risk factors for AKI associated with admission

Correlation coefficients between all variables included in the multivariable model were less than 0.4. Multivariate cause-specific proportional hazards models showed that several variables were risk factors (Table 2). The variables with a multivariate hazard ratio (HR) > 1.2 were dementia, chronic pulmonary disease, rheumatic disease, liver disease, diabetes, hemiplegia or paraplegia, renal disease, any malignancy, mineralocorticoid receptor antagonists, calcium channel blockers, and diuretics. The multivariate variables with HR < 0.9 were female sex, statins, fibrates, high estimated GFR, and physical activity habits.

**Table 2:** Results of cause-specific proportional hazards model

Variable (reference)	Category or unit	Univariate		Multivariate	
		Hazard ratio (95% CI)	*P* value	Hazard ratio (95% CI)	*P* value
Age (0 to < 40 years)	1 year	1.11 (1.11–1.11)	< 0.001	NA	NA
Age	40 to < 50 years	1.80 (0.57–5.72)	0.3	1.67 (0.41–6.82)	0.5
	50 to < 60 years	2,54 (0.81–7.97)	0.1	1.41 (0.35–5.70)	0.6
	60 to < 70 years	3.82 (1.23–11.9)	0.02	1.34 (0.34–5.37)	0.7
	70 to < 80 years	9.07 (2.92–28.2)	< 0.001	1.90 (0.47–7.61)	0.4
	≥ 80 years	28.2 (9.10–87.6)	< 0.001	3.20 (0.80–12.8)	0.1
Sex (male)	Female	0.45 (0.43–0.47)	< 0.001	0.52 (0.49–0.55)	< 0.001
Body mass index	1 kg/m^2^	1.02 (1.01–1.03)	< 0.001	0.97 (0.96–0.98)	< 0.001
Comorbidities					
Peripheral vascular disease (absence)	Presence	2.57 (2.44–2.71)	< 0.001	1.13 (1.06–1.20)	< 0.001
Cerebrovascular disease (absence)	Presence	2.69 (2.56–2.82)	< 0.001	1.10 (1.03–1.16)	< 0.001
Dementia (absence)	Presence	3.95 (3.62–4.30)	< 0.001	1.58 (1.43–1.75)	< 0.001
Chronic pulmonary disease (absence)	Presence	1.74 (1.66–1.83)	< 0.001	1.24 (1.18–1.31)	< 0.001
Rheumatic disease (absence)	Presence	1.91 (1.73–2.11)	< 0.001	1.38 (1.23–1.54)	< 0.001
Peptic ulcer disease (absence)	Presence	2.06 (1.97–2.16)	< 0.001	1.12 (1.06–1.18)	< 0.001
Liver disease (absence)	Presence	1.72 (1.63–1.81)	< 0.001	1.22 (1.15–1.30)	< 0.001
Diabetes (absence)	Presence	4.26 (4.03–4.51)	< 0.001	1.88 (1.76–2.00)	< 0.001
Hemiplegia or paraplegia (absence)	Presence	2.97 (2.53–3.48)	< 0.001	1.25 (1.03–1.52)	< 0.001
Renal disease (absence)	Presence	24.8 (23.7–26.0)	< 0.001	3.36 (3.14–3.59)	< 0.001
Any malignancy (absence)	Presence	2.45 (2.31–2.60)	< 0.001	1.27 (1.19–1.36)	< 0.001
Medications					
ACE inhibitor (no use)	Prescribed	2.68 (2.47–2.92)	< 0.001	1.08 (0.98–1.19)	0.1
ARB (no use)	Prescribed	3.30 (3.15–3.44)	< 0.001	1.14 (1.08–1.21)	< 0.001
MRA (no use)	Prescribed	5.94 (5.45–6.48)	< 0.001	1.26 (1.14–1.40)	< 0.001
CCB (no use)	Prescribed	3.20 (3.06–3.35)	< 0.001	1.36 (1.29–1.44)	< 0.001
β-Blocker (no use)	Prescribed	3.31 (3.10–3.52)	< 0.001	1.11 (1.03–1.20)	0.006
Diuretics (no use)	Prescribed	5.57 (5.30–5.86)	< 0.001	1.31 (1.23–1.40)	< 0.001
SGLT2 inhibitor (no use)	Prescribed	3.41 (2.06–5.67)	< 0.001	1.72 (0.95–3.12)	0.07
NSAIDs (no use)	Prescribed	1.58 (1.51–1.65)	< 0.001	1.02 (0.97–1.08)	0.4
Statin (no use)	Prescribed	1.27 (1.21–1.33)	< 0.001	0.84 (0.80–0.89)	< 0.001
Fibrate (no use)	Prescribed	1.72 (1.55–1.92)	< 0.001	0.83 (0.73–0.95)	0.008
Gout suppressant (no use)	Prescribed	6.64 (6.33–6.97)	< 0.001	1.18 (1.11–1.25)	< 0.001
Result of health checkup					
AST	10 U/L	1.04 (1.02–1.06)	< 0.001	0.98 (0.96–1.00)	0.12
Estimated GFR	5 mL/min/1.73 m^2^	0.62 (0.61–0.62)	< 0.001	0.73 (0.73–0.74)	< 0.001
Questionnaire response					
Physical activity habits (no)	Yes	0.69 (0.66–0.73)	< 0.001	0.79 (0.75–0.83)	< 0.001

The eGFR was calculated as follows: 194 × creatinine^−1.094^ × age^−0.287^ (× 0.739 [if women]). The number of samples used for the complete case analysis was 541,014, and the number of events (AKI) was 6560 (86,391 samples were removed owing to missing data)

*NA* not applicable, *ACE* angiotensin-converting enzyme, *ARB* angiotensin receptor blocker, *MRA* mineralocorticoid receptor antagonist, *CCB* calcium channel blocker, *SGLT2* sodium-glucose transporter 2, *NSAIDs* nonsteroidal anti-inflammatory drugs, *AST* aspartate transaminase, *GFR* glomerular filtration rate

The sensitivity analysis taking into account the interaction terms for statins (cerebrovascular disease and peripheral vascular disease) showed a similar result to that for just statins (HR 0.85, 95% confidence interval 0.79–0.91).

## Discussion

We found several factors related to AKI associated with admission. In addition, the incidence of AKI in the general population was estimated. Our study is the first investigation to examine these issues using claims data from an Asian population.

Multivariate analysis results suggested that the administration of statins and physical activity habits are novel protective factors against AKI.

Robust evidence supports the preventive effect of statins on cardiovascular and cerebrovascular disease [17]. Statins are also suggested to benefit kidney disease via mechanisms such as anti-inflammatory, anti-oxidative, and endothelial protective effects [18]. Regarding the association between kidney disease and statins, statins showed beneficial effects in lipid management in patients with chronic kidney disease (CKD) [17, 19]. There is insufficient evidence of an association between statins and AKI. Existing studies suggest that statins both increase the risk [20, 21] and do not affect the risk [22, 23] of AKI. In previous studies unable to indicate the effectiveness of statins, the severity of the underlying disease might have hindered the efficacy of statins [17]. Given that our study was conducted in a healthy general population, we would expect to detect the therapeutic effectiveness of statins.

Historically, healthy lifestyles, including physical activity habits, have prevented several diseases. Studies using data from the Japanese Specified Health Checkups, similar to ours, have shown the association between healthy lifestyles, including physical activity habits, and CKD [24], diabetes, and hypertension [25]. In addition, large prospective studies have shown that lifestyle modifications, including physical activity habits, can prevent cardiovascular disease [26]. Although no previous studies have examined the association between AKI and physical activity habits, a meta-analysis suggested that frailty is a risk factor for AKI [27]. Considering the results of previous studies and our current data, it is suggested that improving physical activity habits may contribute to the prevention of AKI.

Other variables associated with AKI in our study were approximately consistent with those in previous studies [5, 28–31]. In contrast with previous reports [30], nonsteroidal anti-inflammatory drugs and angiotensin-converting enzyme inhibitors were not a significant risk factor. Although fibrates are generally known to affect kidney function [33], we obtained the contrary result. We assume that these discrepancies are due to the avoidance of prescribing these drugs to patients at high risk for kidney injury.

The incidence of AKI estimated in this study (251 per 100,000 person-years) differs somewhat in comparison with previous studies. Several prospective observational studies in the general population have reported the incidence of severe AKI requiring dialysis as 13–14 cases per 100,000 person-years [33, 34], one of which was conducted in the same region as the present study (Shizuoka, Japan) [34]. Another cohort study in a population with normal renal function reported the incidence of AKI requiring hospitalization as 100 per 100,000 person-years, of which 10 cases in 100,000 were severe AKI requiring dialysis [35]. These results estimate that severe AKI accounts for less than 10% of all AKI. Our study includes patients with impaired baseline renal function and estimated AKI incidence for all severities. Therefore, the higher incidence of AKI than previously reported elsewhere is reasonable. Additionally, we consider that the present results provide useful epidemiological information about AKI in the general population, especially among older people in Japan. Risk factors for AKI are generally similar between our cohort and others, assuming that the populations of these cohorts are comparable. Based on the aforestated, we consider that we extracted AKI from the administrative claims database with a reasonable level of accuracy. Therefore, we were able to explore factors associated with AKI and estimate its incidence in this reliable population.

Although we constructed a linear risk prediction model for the overall population, recent reports have shown that there is heterogeneity in predictive outcomes between individual subgroups in the prediction model and that interactions between risk factors should also be taken into account [36]. The present study attempted to identify risk factors that are independent in nature from linear regression analysis and to suggest the existence of intervening factors that contain bias but have large effects. Moreover, no causal effect estimates for individual factors were made. Therefore, we did not assess predictive performance or examine interactions as a potential risk and confounding factors. Despite these limitations, we believe that the presentation of modifiable factors from simple studies such as ours can help prevent disease.

There are some other limitations to this study. First, only ICD-10 codes were used to define AKI because the data concerning urinary output and changes in renal function around the hospitalization period were unavailable. To preserve the validity of the study, we used ICD-10 codes in accordance with previous studies [10–12]. Nevertheless, as a limitation of the coding-based definition of AKI, AKI extraction in our cohort had high specificity, but low sensitivity, and could have been biased to severe cases [37]. Second, we could not use variables not included in the user database; thus, potential risk factors may not have been investigated. We included the major known risk factors for AKI (comorbidities and medications) in multivariate analysis to explore the circumstances as much as possible and conducted a sensitivity analysis with consideration of interaction terms. Third, because the SKDB contains only a Japanese population, caution should be exercised in extrapolating this study’s results to other ethnic groups. Fourth, our dataset may have a time discrepancy between the prescription of medication as risk factors and the onset of AKI. Most medications are regularly prescribed for chronic disease. Therefore, the effect of this limitation is considered to be relatively small. In contrast to previous studies [28], non-steroidal anti-inflammatory drugs were not significant in the multivariate analysis. These types of medications with a shorter prescription duration may have been more affected by this limitation.

## Conclusions

The factors associated with AKI and the incidence of AKI associated with hospital admission in the general Japanese population are highlighted. This study generates the hypothesis that statins and physical activity habits are novel protective factors for AKI, although these exploratory results need to be validated in prospective trials. This study of AKI promises to provide essential insights into its etiology and prevention strategy.

## Supplementary Information

Below is the link to the electronic supplementary material.

Supplementary file1 (DOCX 17 KB)
